# Feasibility of a new intervention addressing group-based balance and high-intensity training, physical activity, and employment in individuals with multiple sclerosis: a pilot randomized controlled trial

**DOI:** 10.3389/fresc.2023.1258737

**Published:** 2024-01-08

**Authors:** Ellen Christin Arntzen, Tonje Braaten, Hanne Kristin Fikke, Britt Normann

**Affiliations:** ^1^ Faculty of Nursing and Health Sciences, Nord University, Bodø, Norway; ^2^ Department of Physiotherapy, Kongsgården Physiotherapy, Bodø, Norway; ^3^ Department of Community Medicine, UiT The Arctic University of Norway, Tromsø, Norway; ^4^ Department of Physiotherapy, Nordland Hospital Trust, Bodø, Norway

**Keywords:** multiple sclerosis, sensorimotor, feasibility, balance, walking, high-intensity, physical activity, employment

## Abstract

**Background and purpose:**

Impaired sensorimotor function, reduced physical activity and unemployment are common challenges in persons with multiple sclerosis (pwMS), even when disability is low. CoreDISTparticipation is a new, multidisciplinary intervention delivered across healthcare levels systematically addressing these elements. This study primarily aimed to evaluate the feasibility of CoreDISTparticipation in terms of process, resources, management, and scientific outcomes. The secondary aim was to evaluate initial efficacy in terms of possible short-term effects compared with the usual care on barriers to employment, balance, walking, health-related quality of life (HRQoL), and physical activity.

**Methods:**

This assessor-blinded prospective pilot randomized controlled trial included 29 pwMS [Expanded Disability Status Scale (EDSS): 0–3.5] randomly allocated to the intervention group (CoreDISTparticipation) (*n* = 15) or usual care (*n* = 14). CoreDISTparticipation consists of three phases: (1) hospital outpatient clinic: MS nurse work-focused session and physiotherapist exploring balance; (2) municipality: a digital meeting with pwMS, employer, MS nurse, and physiotherapist addressing employment and physical activity, 4 weeks indoor CoreDIST balance training (60 min × 2/week); and (3) 4 weeks outdoor CoreDIST balance training and high-intensity running/walking (60 min × 2/week). Assessments were undertaken at baseline and at weeks 6 and 11. Primary feasibility metric outcomes were the reporting of process, resources, management, and scientific outcomes. Efficacy measures included evaluation of the Multiple Sclerosis Work Difficulties Questionnaire-23 Norwegian Version (MSWDQ-23NV) and 6 Minute Walk-test as well as the Trunk Impairment Scale-modified Norwegian Version, Mini-Balance Evaluation Systems Test (Mini-BESTest), Multiple Sclerosis Walking Scale-12, Multiple Sclerosis Impact Scale-29 Norwegian Version (MSIS-29NV), ActiGraph wGT3x-BT monitors, and AccuGait Optimized force platform. The statistical analyses included repeated-measures mixed models performed in IBM SPSS Version 29.

**Results:**

The primary feasibility metric outcomes demonstrated the need for minor adjustments in regard to the content of the intervention and increasing the number of staff. In regard to the efficacy measures, one person attended no postintervention assessments and was excluded, leaving 28 participants (mean EDSS: 1.8, SD: 1). The mean percentage employment was 46.3 (SD: 35.6) and 65.4 (SD: 39.3) in the CoreDISTparticipation and usual care group, respectively. No between-group differences were found. MSWDQ-23NV demonstrated a within-group difference of 5.7 points from baseline to Week 11 (*P* = 0.004; confidence interval: 2.2–9.3). Mini-BESTest and MSIS-29NV demonstrated within-group differences. The study is registered in ClinicalTrials.gov (Identifier: NCT05057338).

**Discussion:**

The CoreDISTparticipation intervention is feasible to support pwMS when the identified feasibility metric outcomes in regard to process, resource, management, and scientific outcome metrics are adjusted to improve feasibility. Regarding efficacy measures, no between-group differences were detected; however, within-group differences in barriers to employment, balance, and HRQoL were detected for the CoreDISTparticipation group. A larger comparative trial is needed to explore between-group differences and should accurately and precisely define usual care and address the identified limitations of this study.

## Introduction

Multiple sclerosis (MS) is a chronic, neurological disease (1) that affects adults and children at any age, with a mean age of diagnosis of 32 years (2). The disease often follows an unpredictable and fluctuating course with accumulation of sensorimotor disturbances, balance and walking problems, fatigue, and cognitive problems (3–7). All these challenges, which are common even in the early stages, are associated with low levels of physical activity, impaired health-related quality of life (HRQoL), unemployment or reduced positions, and are accompanied by substantial personal burdens and societal costs (8, 9). Of Concern, only approximately half of individuals living with MS and mild disability seem to be meeting the current physical activity guidelines (10), and unemployment is reported in 55%–70% of persons with MS (pwMS) (11–13). Globally, 43% of pwMS leave their jobs within the first 3 years after diagnosis, and 70% quit within 10 years (9). These reports demonstrate that physical activity and employment are of major importance for people with MS (14) and should be monitored and addressed from the very start of the diagnosis (15).

Factors associated with pwMS staying employed are having low levels of fatigue and few mobility-related symptoms (12, 14, 16). Physical activity, physiotherapy, and exercise can reduce fatigue (17, 18), improve balance, walking (19, 20), and HRQoL (18, 21), and may also improve neuromuscular and physical functioning in pwMS (22). It is recommended to start exercising from the start of the diagnosis (23). This early phase, when disability is often mild and neuroplasticity is optimal, is the best window of opportunity to optimize sensorimotor function (22, 24), create good habits with regard to physical activity, and a basis for optimizing sustained employment. Physical activity levels lower than the recommended minimum of 150–300 min of moderate training, exercise, or physical activity per week (23, 25, 26) are common both in the MS population in general (27, 28) and in individuals with mild disability (29). The self-reported dose and intensity of physical activity have even decreased during the COVID-19-pandemic (30).

To meet these complex challenges regarding sensorimotor function, physical activity, and work, we have developed a comprehensive multidisciplinary intervention and pathway delivered across healthcare levels, targeting the promotion of balance, walking, physical activity, and work participation. This new, individualized, group-based intervention, entitled CoreDISTparticipation, is built on GroupCoreDIST (31), which has previously appeared to be feasible (32), effective in improving balance, trunk control (29), and walking (33), and meaningful among ambulant pwMS (34) and physiotherapists (35–37). The GroupCoreDIST intervention focused on prerequisites for postural control and balance and was undertaken in an indoor environment for 60 min, three times per week for 6 weeks. CoreDIST stands for the coordinated relationship between the proximal and distal areas of the body, as core/trunk muscle activation coordinated with activity in the extremities is important for postural control during balance, walking, and daily activities (38). Furthermore, CoreDIST emphasizes elements of importance for motor learning, such as high dose (D), dual task (D) individualization (I), (high) intensity (I) and insights/meaning (I), as well as addressing underlying prerequisites for postural control, such as somatosensory function (S), selective movement (S), muscle length, and advanced balance challenges in a task-oriented training format (T). The new intervention, CoreDISTparticipation, links specialist and municipal healthcare services and includes the person with MS and their employer. The group training has been expanded to include 4 weeks of indoor GroupCoreDIST training followed by 4 weeks of outdoor training. The new elements in CoreDISTparticipation are as follows: (1) an assessment with a physiotherapist at the hospitals MS outpatient (MS-OP) clinic assessing balance and potential for change in sensorimotor function, (2) a work-related session with an MS nurse at the MS-OP clinic, (3) a digital multidisciplinary meeting with the pwMS, their employer, the municipal physiotherapist, and the MS nurse, (4) outdoor CoreDIST sessions integrating balance and high-intensity interval training, and (5) digital home-exercise videos. To the best of our knowledge, follow-ups integrating work, physical activity, and combinations of high-intensity training and specific sensorimotor functions have not been previously explored. Therefore, the primary aim of this study was to evaluate the feasibility of the new intervention, CoreDISTparticipation, in terms of process, resources, management, and scientific outcomes. The secondary aim was to evaluate initial efficacy in terms of possible short-term effects on barriers to work, walking distance, physical activity, balance, and HRQoL. Evaluating various feasibility metrics in preparation for a subsequent large-scale study is in line with current recommendations (39, 40). We posed the following research question: *What are the feasibility and short-term preliminary effects of CoreDISTparticipation compared with the usual care on barriers to employment, balance, walking, quality of life, and physical activity in individuals with MS having lower levels of disability?*

## Materials and methods

### Trial design

This two-armed prospective, assessor-blinded pilot feasibility randomized controlled trial (RCT) included 29 individuals with mild to moderate disability due to MS measured by the Expanded Disability Status Scale (41) (EDSS ≤ 3.5).

### Ethics

The study was approved by the Regional Committee for Medical Research Ethics, North Norway (Grant number 174837) and the Local Ethical Committee at the Nordland Hospital Trust (NLSH) (Project number 209). It was registered in ClinicalTrials.gov (Identifier: NCT05057338) and was conducted in accordance with the Helsinki Declaration. All participants provided written informed consent prior to inclusion. The study was funded by the Northern Norwegian Regional Health Authorities (Grant number 174837). The funder played no role in the design, conduct, or reporting of the study. The CONSORT guidelines (42) were followed throughout the study.

### Context of the study

**Figure 1:**
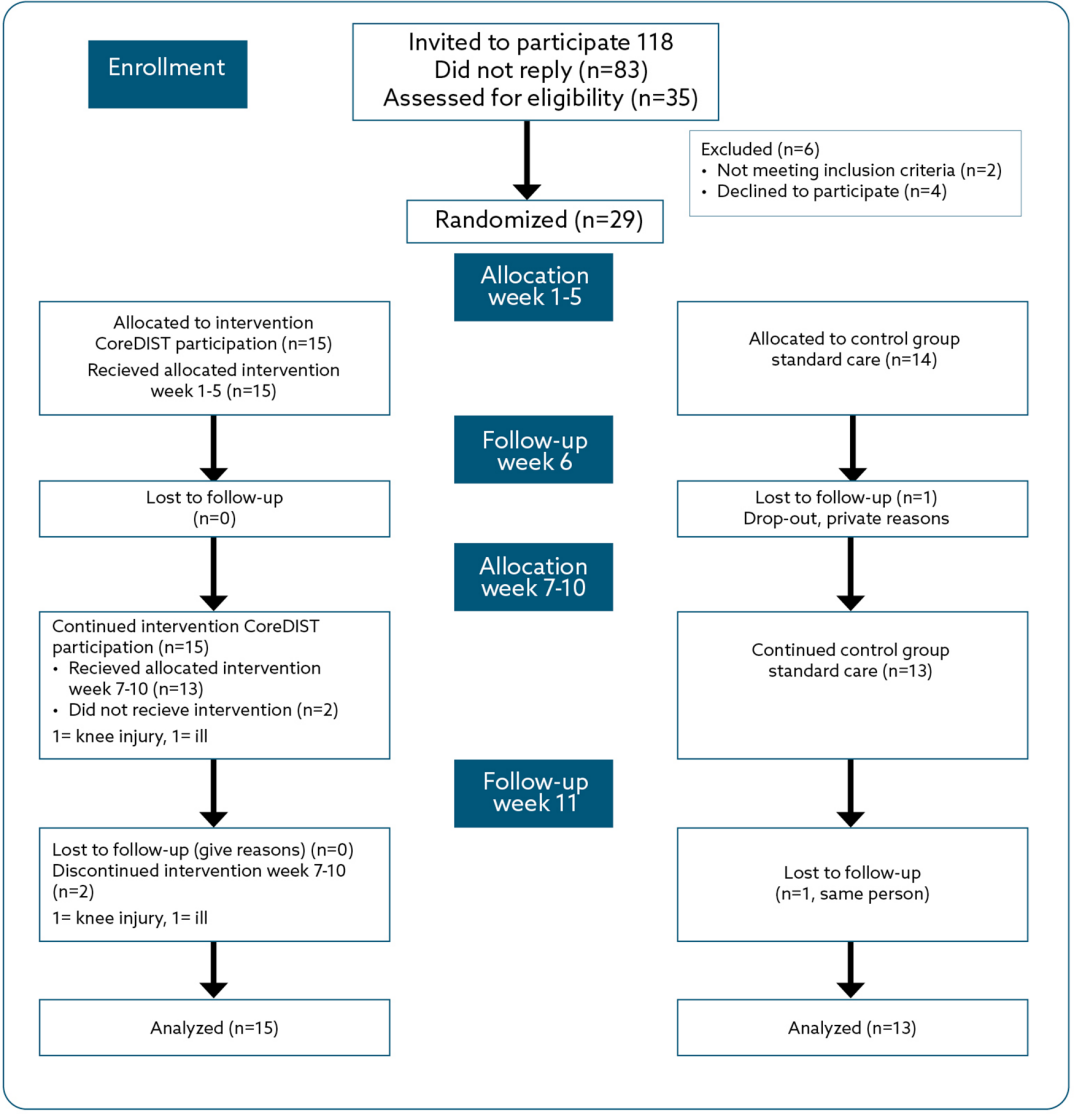
Flow chart for enrolment, allocation, and follow-up for the CoreDISTparticipation and usual care groups.

The study was conducted between August and November 2021 at NLSH's (regional hospital) MS-OP clinic in cooperation with physiotherapists in two municipalities (50,000 and 10,000 inhabitants, respectively) (Figure 1, flow chart). The project group consisted of three user representatives from the Nordland MS Association, an MS nurse, a neurologist, and three physiotherapists.

### Preparations and procedures

One physiotherapist, a specialist in neurological physiotherapy, who was adequately trained in the assessment procedures and blinded to group allocation, performed all assessments. One physiotherapist at the MS-OP clinic conducted the first assessment. An experienced MS nurse took part in the digital meetings. Four physiotherapists, two specialists in neurological physiotherapy, and two generalists led the training groups. These followed 2 days of practical and theoretical training and 2 h of digital education in the CoreDISTparticipation program. Guidelines for the first assessment, the MS nurse meeting regarding work, and the multidisciplinary digital meeting were developed by the project group. The user representatives took part in the planning, implementation, discussion of results, and evaluation of the study.

### Participants and recruitment

In July 2021, written information regarding the study was sent by post from the MS-OP clinic to the pwMS who were registered at the Nordland Hospital Trust, lived in the two selected municipalities, and had EDSS ≤ 3.5 (*n* = 118). One reminder was sent to ensure maximum patient enrolment, and 35 individuals replied with signed informed consent. We had limited information regarding how many of the 118 invited persons were employed; therefore, we expected a low response rate. Enrolment started in August 2021, and follow-up assessments were completed in November 2021, organized by the MS-OP clinic. At enrolment, the EDSS values of all participants were recorded from their hospital journals. The inclusion criteria were a diagnosis of MS according to McDonald's criteria (43), EDSS ≤3.5 (no disability/fully ambulatory with moderate disability), 18 years or older, and being employed (10%–100%). The exclusion criteria were pregnancy at enrolment, exacerbation within 2 weeks prior to enrolment, and other serious conditions compromising balance, walking, or work capacity, such as recently surviving a stroke. Of the 35 participants who consented to participate, six were excluded based on the following: four withdrew their consent based on lack of time or other personal reasons, one had an EDSS higher than 3.5, and one turned out not to have MS. This left a sample of 29 pwMS.

### Randomization

After baseline assessments, the participants were randomized to CoreDISTparticipation (intervention group) or usual care (control group) in a 1:1 relationship. We stratified for municipality to ensure possibilities for group training using the Research Electronic Data Capture Tool (performed by ECA), supported by the Clinical Research Department, University Hospital of Northern Norway.

### Interventions: CoreDISTparticipation and usual care

Both groups continued their regular medical treatment and were encouraged to stay employed and to be physically active.

CoreDISTparticipation is a further development of GroupCoreDIST developed by Normann and Arntzen and I-CoreDIST developed by Normann, Sivertsen, and Arntzen (32, 44). Norman and Arntzen further developed the CoreDISTparticipation, and S.S. Haakonsen Dahl contributed to developing the outdoor training. The TIDieR author tool has been used in the development of the new intervention (45). The CoreDISTparticipation consists of three phases and is further described in the Appendix.

The usual care group continued routine consultations at the MS-OP clinic with the usual follow-up in the municipality, including physiotherapy, physical activity, or other follow-up.

### Feasibility metric outcome measures and registrations

The primary feasibility metric outcome measures were registered by the researchers, assessors, clinicians, and participants in the study. These included (1) *process*: recruitment rates, retention rates, appropriateness of eligibility criteria, participants compliance with protocol, indoor and outdoor sessions, digital meetings with the MS nurse, digital multidisciplinary meetings, and participants reactions to assessment; (2) *resources*: time from sending out invitations to response, time for clinician to learn the intervention, time for assessment of outcome measures and for patients to fill out questionnaires, budget, intervention sustainability within the proposed setting, staff training needs, and equipment access; (3) *management:* personnel, data management, resource site capacity, assessment capacity, MS outpatient clinic MS nurse and physiotherapist capacity, including municipal physiotherapists, equipment usage for digital meetings, meeting links sent from the hospital, data processing time, and software appropriateness; and (4) *scientific metrics*: safety, outcome measures, content of intervention of the indoor and outdoor training, dose and intensity of the intervention, fidelity of the intervention (therapists), patients’ acceptance of the intervention and tolerance to the protocol, and potential participant bias. All metrics were discussed in the research group during and after the study was completed.

At baseline, demographic characteristics were registered: age, gender, marital status, years of education, current employment status, how much the participants wanted to work if the job was perfectly arranged for them, type and duration of MS, EDSS, smoking, and medications. The assessments were undertaken by a blinded, independent tester at baseline, Follow-up 1 (Week 6), and Follow-up 2 (Week 11) (Figure 1, flow chart). The secondary feasibility metrics were the efficacy measures that were considered to be the most relevant for the aim of the captured employment-related barriers through the Multiple Sclerosis Work Difficulties Questionnaire-23 Norwegian Version (MSWDQ-23NV) and walking capacity [6-Min Walk Test (6MWT)]. The MSWDQ-23NV is a 23-item questionnaire that measures how frequently a pwMS perceives psychological/cognitive (11 items), physical (eight items), and external barriers (four items) related to their current or latest job. It is scored on a five-point scale (best score = 0) (46). The MSWDQ-23NV has recently been translated into Norwegian and is currently undergoing research evaluation for validity and responsiveness. The 6MWT is a valid and reliable outcome in pwMS that measures the distance within 6 min of walking in a 25 m long hallway (47). The minimal clinically important difference for pwMS is calculated to be 19.7 m for improvement (48).

The other efficacy outcome measures were the Trunk Impairment Scale-modified Norwegian Version (TIS-modNV), which records dynamic trunk control and sitting balance. It has a 0–16 rating scale, is valid for individuals with stroke, has been frequently used in the MS population, and is currently being validated (ClinicalTrials.gov Identifier: NCT05057338). The Mini-Balance Evaluation Systems Test (Mini-BESTest) measures pro- and reactive balance in sit to stand, standing, and walking. There are 14 items, each scored on a three-point scale with a top score of 28. The Mini-BESTest is valid and reliable for pwMS (49). The AccuGait Optimized force platform measures postural control in the form of symmetry/asymmetry of weight-bearing in standing, postural sway of centre of pressure (COP) as the participant stands on the platform with feet close together and at hip-width with eyes open and closed. Data on COP displacements in centimetres were collected for 30 s with a frequency of 50 Hz in the domains eyes open and eyes closed, and root mean square (RMS) values of the COP displacements were calculated (50). ActiGraph wGT3x-BT monitors measure physical activity levels (inactive, light, moderate, vigorous) and number of steps (51). The activity monitor was worn in a belt around the waist for seven consecutive days after all assessment points. The ActiGraph is an objective measure of community ambulation and physical activity in pwMS (52).

The EQ-5D-3l (European Quality of Life 5-Dimension-3-Level) measures self-perceived HRQoL regarding five domains, each with three items, and a VAS scale (0–100) recording perceived health (53). The MS Impact Scale 29-Norwegian Version (MSIS-29NV) measures self-perceived physical (20 items) and psychological (nine items) impact of MS on HRQoL recorded by a five-point scale (54). The MSIS-29NV is valid and reliable in Norwegian pwMS (55). Eight points is considered a minimal clinically important difference in pwMS (56). The MS Walking Scale-12 (MSWS-12) measures self-perceived limitations in walking due to MS on 12 items with a scale of 1–5. The MSWS-12 is valid and reliable in pwMS (57, 58). A change of −0.7 points from the patient perspective and −10.7 points from the therapist perspective is considered clinically meaningful in pwMS having mild to moderate disability (EDSS ≤ 4) (59).

After the in- and outdoor trainings, the participants scored the Borg scale regarding intensity of the training. During the outdoor training, some participants wore pulse belts and watches (some wore their own, and the municipal physiotherapists lent out three watches) to monitor the intensity of the training. As these watches were neither calibrated nor validated and not worn by all participants, the results were used only to monitor the trainings.

### Sample size

The sample size was based on prior literature where 20–40 participants are recommended for pilot studies to enable estimates of standard deviations to calculate sample size for subsequent large-scale studies (60).

### Data and statistical analysis

IBM SPSS version 29 (IBM, Armonk, NY, USA) was used, and the intention-to-treat principle was applied for all analyses. Descriptive statistics were used to clarify the clinical and demographic characteristics of the sample, baseline measurements, and recordings at all time-points. Linear regression models were applied to examine the score differences between groups adjusted for baseline score in all outcomes at each follow-up. Linear mixed models were used to examine between-group differences over time and overall between-group differences at follow-up adjusted for baseline scores, where the term overall refers to the mean of the outcome values at Weeks 6 and 11. We used the Bayesian Information Criterion to select the appropriate statistical models. The model assumptions were assessed by residual plots and considered sufficiently fulfilled. All time-points were included in one model. The results from the primary outcomes will serve as a basis for sample size calculations and for adjustments of CoreDISTparticipation when planning a subsequent large-scale study.

## Results

The clinical and demographic characteristics for all participants are presented in Table 1. Out of 118 pwMS contacted, 35 responded with informed consent, which is a response rate of 34%. After screening in relation to inclusion and exclusion criteria, a sample of 29 pwMS took part. Regarding trial completion, one person in the control group did not attend any follow-up assessments and hence was excluded, which meant that 28 pwMS completed the whole trial. The primary feasibility metrics in terms of process, resources, and management are presented in Table 2. The attendance was high for indoor GroupCoreDIST, with an 85% attendance rate (mean 6.8 sessions of eight possible sessions per person). The attendance for outdoor sessions was moderate and ranged from 0 to 8 sessions, with a mean of 4.6 sessions per person and altogether 57.3% attendance. There was a 100% attendance for the digital meetings with the MS nurse and the multidisciplinary meetings. There were no reports of new relapses or adverse events during assessments. During high-intensity running/walking, three adverse reactions were reported; a hamstring strain, an ankle strain, and increased dizziness. A fourth person felt unsecure about reaching a high pulse because of heart medications. No new relapses were reported during the study period.

**Table 1:** Baseline clinical and demographic characteristics of the participants in the CoreDISTparticipation and usual care group as measured by means, mean percentage, and standard deviation.

Baseline characteristics	Usual care *n* = 13	CoreDISTparticipation *N* = 15
Age	50.5 (SD: 10.8)	47.6 (SD: 6.0)
Height (cm)	172.0 (SD: 7.9)	170.6 (SD: 8.6)
Weight (kg)	73.6 (SD: 13.1)	73.9 (SD: 14.1)
Gender		
Women	9 (69%)	12 (80.0%)
Men	4 (31%)	3 (20.0%)
Smoker		
Yes	0	2 (13.3%)
No	13 (100%)	13 (86.1%)
Type of MS		
Relapsing remitting	11 (84.6%)	15 (100%)
Primary progressive	2 (15.4%)	0
EDSS	1.7 (SD: 1.1)	1.8 (SD: 0.9)
Years since diagnosis	12.0 (SD: 11.2)	10.4 (SD: 7.8)
Currently employed		
Yes	12 (92.3%)	12 (80.0%)
No (did not work the previous month)	1 (7.7%)	3 (20.0%)
Current employment percentage (mean)	65.4 (SD: 39.3)	46.3 (SD: 35.6)
Current sick leave percentage (mean)	25.4 (SD: 41.2)	16.7 (SD: 35,4)
Current disability pension percentage (mean)	14.6 (SD: 23.0)	30 (SD: 33.2)
Preferred work percentage if the job was adjusted to their needs (mean)	83.8 (SD: 22.6)	72.7 (SD: 26.3)

**Table 2:** Primary feasibility metrics in terms of process, resources, management, and safety with reflection on changes needed to improve for a future trial.

Rational	Classification	Success factors/barriers	Type of examination	Changes and preparations for the large-scale study
Process	Recruitment rates	Low response rate (34%) to invitation to take part in research	The low response rate was expected as approximately 50% of pwMS are not working, and therefore were not within the inclusion criteria	Seventeen municipalities will be included to gain the appropriate sample size of 114 participants. We will have one municipality as backup in case of low response MS charitable organizations and user representatives will be more involved in the promotion of the study The information and informed consent will be sent by email
	Retention rates	High intervention group: 15/15 and control group: 13/14	Results	We will continue to maintain regular contact with participants through assessments. Participants will be followed for a longer time by a physiotherapist and by the welfare system
	Appropriateness of eligibility criteria	Adequate The sample was representative for the target population of employed pwMS	Results compared with a survey conducted in the same area exploring pwMS	Individuals with EDSS 0–4 will be included to target a larger sample of employed pwMS
	Participants compliance with protocol Indoor sessions	A mean compliance of 85% for indoor sessions; mean 6.8 sessions of eight possible sessions per person	Results: registrations by the municipal physiotherapist	Indoor sessions will change to once per week
	Participants compliance Outdoor sessions	Moderate 57.3% attendance Mean 4.6/8 sessions per person	Results: registrations by the municipal physiotherapist	Outdoor sessions: change to once per week (1× indoor and 1× outdoor per week). Outdoor high-intensity assessment will be part of the first examination at the municipal physiotherapist
	Participants compliance Digital meetings with MS nurse	100% attendance	Results registrations by the MS nurse	Digital attendance feasible for the participants
	Participants compliance Digital multidisciplinary meetings	100%	Results registrations by the municipal physiotherapist	Digital attendance feasible for the participants
	Participants reactions to assessment	Two persons reported performance anxiety during assessments	Reports from assessor	Increase information given before assessments regarding the aim of each outcome measure
Resources	Time from sending out invitations to response	All participants were included within 1 month	Reports from researchers	Continue good preparations such as working closely together with user representatives and the MS federation, promotion on social media, digital, and physical meetings
	Time for clinician to learn the intervention	Two days physical and 2 h digital meeting that from the clinician's point of view was reported to be appropriate	Reports from researchers and conversations with clinicians	Adequate time if physiotherapists are preparing through self-study
	Time for assessment of outcome measures and for patients to fill out questionnaires	60 min per person; assessors reported it to be appropriate although a bit stressful	Assessor’s experience	Questionnaires will be sent out digitally to gain more time
	Budget	The study was funded and there was enough money to support all parts of the study	Project leader’s experience	Funding was adequate
	Intervention sustainability within the proposed setting	4 weeks indoor and 4 weeks outdoor sessions in the municipality was adequate	Municipal physiotherapists experience	This relatively short period was adequate although the group follow-up will in the large-scale study last for 6 weeks, and a period with digitally supported home training will be added
	Staff training needs	Training of assessor for 4 h as the assessor knew all outcome measurements in advance and was an experienced assessor	Researcher and assessors experience	One day is needed in order for the two assessors to gain reliability of the outcome measures.
	Equipment access	ActiGraphs: takes long time to send back to researchers by post Balance platform: large platforms that are not so easy to transport Equipment for the Mini-BESTest: the inclined board and balance cushion were not so easy to transport	Assessors experience	ActiGraphs: calculate 2 weeks for participants to send back to researchers by post Balance platform: one platform is needed at each test location Equipment for the Mini-BESTest: access to equipment at every test location.
Management	Personnel	Day-to-day management: one person Assessor: one person This was adequate for the pilot study, although in some periods the study took all focus	Assessor and researcher’s experience	Three to four persons are needed for management of the large-scale study Two assessors and two backup assessors are needed
	Data management	Researcher and statistician were adequate for the pilot study	Researcher’s experience	Three persons for data management are needed + a statistician and a statistician with special expertise in social economics
	Resource site capacity: assessment	Adequate Important to plan a long in advance of assessments	Researcher’s and assessor’s experience	At least two assessment sites are needed. Time for assessments needs to be planned long in advance
	Capacity: MS outpatient clinic MS nurse	MS nurse had low capacity and felt the extra job a burden	Conversations with MS nurse and leaders at the MS outpatient clinic	The MS nurse’s role in the project should be conducted by the welfare system
	Capacity: MS outpatient clinic physiotherapist	Adequate Physiotherapist and department were motivated Vulnerable with only one physiotherapist in case of illness	Conversations with physiotherapist and department leaders	At least two physiotherapists at each MS outpatient clinic should learn the intervention and take part in the project
	Capacity: Municipal physiotherapists	Adequate Private practice physiotherapists have easier access to the particular population in a normal (non-trial) setting	Conversations with physiotherapists and leaders in the municipalities	Private practice physiotherapists should be included as they have easier access to the particular population in a normal (non-trial) setting
	Equipment usage for digital meetings, meeting links sent from the hospital	The meeting links did not work well, especially if there were many persons present at the meetings.	Conversations with physiotherapists in the municipality, the MS nurse, and persons with MS	Up-to-date meeting links will be tested in advance sent from the welfare system
	Data processing time	Assessments were completed on November 2021 and submission of paper was on July 2023 because of delay in punching data and calculating results. The researchers had to prioritize other jobs and the data processing therefore took a longer time than expected. A statistician was needed and joined in the team in the autumn of 2022	Researcher’s and assessor’s experience	At least three persons with not so many other tasks are needed. One statistician and one specialist in social economics are needed to speed up data processing time
	Software appropriateness	RedCap for randomization and creating the database and SPSS for calculation of results worked very well	Researcher’s and assessor’s experience	A digital solution for the questionnaires is needed. RedCap will be used
Scientific	Safety	No adverse events due to assessments No reports of relapses during the training. There were three reports of adverse reactions during high-intensity running/walking; a hamstring strain, an ankle strain, and increased dizziness In addition, one person felt unsecure to reach high pulse because of heart medications	Researcher’s, assessor’s and municipal physiotherapist’s reporting. The adverse reactions were taken care of by the municipal physiotherapist. The hamstring and ankle strains were treated acutely within the PRICE principle and advice. No further treatment was needed. The increased dizziness was assessed by the municipal physiotherapist and advice was provided. One person had a known heart condition and was worried to conduct the high-intensity training. This person was informed that high-intensity training is safe; however, chose to maintain a moderate intensity during the sessions	High-intensity training should be tested during the clinical assessment by the municipal physiotherapist More variation in high-intensity activities should be offered, for instance, squats and upper limb exercises with high intensity instead of running Aids should be offered, for instance, Nordic walking sticks Physiotherapists should have access to ice and compression bandages at all sessions
	Outcome measures	MSWDQ-23NV provided promising results. The questionnaire is translated to Norwegian and validated (paper will be submitted in November 2023). Responsiveness values need to be calculated The 6MWT demonstrated promising results; however, it does not reflect the intervention perfectly	Researcher`s discussions	The MSWDQ-23NV will continue as a primary outcome as it reflects the main aim of the intervention. The validation paper needs to be published and responsiveness values calculated before the large-scale study ActiGraphs will be a new primary outcome as number of steps and physical activity levels reflect the aim of the study better than the 6MWT
	Other outcome measures	The other outcomes worked well, although there is a need to explore fatigue more in detail as this is a well-known reason for quitting a job	Researcher’s discussions	The fatigue severity scale will be added as a secondary outcome. Other than this the other outcomes will continue
	Content of intervention Indoor training	Already well established with good results	Researchers, clinicians, and prior effect study	Continue content
	Content of outdoor training	Worked well for most participants, a need for more individual adjustments in the high-intensity training	Experiences from clinicians, results from structured interviews in another CoreDISTparticipation study	Adjustments needed for the high-intensity training. There is a need for alternative exercises while standing that will increase intensity, and a need to include the possibilities to walk or run with walking aids
	Dose of intervention	2× per week for 8 weeks was adequate. Participants were encouraged to conduct home exercises from online videos and to be more physically active in daily life There was no assessment regarding dose of home exercises	Researchers’ experiences and results from another CoreDISTparticipation study	Assessment regarding dose of home exercises and an increased focus on behavioural change are needed
	Intensity of intervention	The intensity was measured by the Borg scale after each training. The results demonstrated that most participants showed low intensity, and some much too low (those who had trouble moving fast enough because of physical symptoms). Some used/wore a pulse watch. The physiotherapist reported that it was easier to adjust the intensity level for these participants	Results and reports from the physiotherapists	Intensity needs to be measured by pulse watches to secure that high intensity is reached
	Fidelity of the intervention (therapists)	The physiotherapists registered which exercises were used for the indoor training. The program for the outdoor sessions was set by the researchers for each of the 4 weeks of outdoor training. A set of potential adjustments was described by the researchers in the protocol. No descriptions of individual adjustments were registered	Researchers’ discussion and results	Registration of exercises is needed. The physiotherapists need to learn the exercises in detail
	Patients’ acceptance of the intervention and tolerance to the protocol	The indoor sessions were well tolerated by all participants and the outdoor sessions were well tolerated by most of the participants who attended the training. Some injury and increasing symptoms were registered during the high-intensity training	Results from another CoreDISTparticipation study	The high-intensity training needs adjustments (see “safety”)
	Potential participant bias	The participants reflected the MS population	Results	No adjustments needed

The main findings were that the primary feasibility metrics were adequate. However, there is a need for adequate access to equipment at all test locations and to increase the number of personnel especially with regard to data management. The MS nurse reported a lack of capacity and inadequate software for digital meetings. In addition, the content of the outdoor sessions would benefit from revision. These details are presented in Table 2.

The efficacy outcome measures in regard to short-term effects are presented in Table 3. There were no differences between the CoreDISTparticipation and usual care group with regard to clinical and disease characteristics at baseline. The EDSS levels were low in both groups, with a mean of 1.7 (SD: 1.1) in the intervention group and 1.8 (SD: 0.9) in the control group. The mean percentage of employees was 46.3 (SD: 35.6) in the CoreDISTparticipation group and 65.4 (SD: 39.3) in the usual care group the previous month. When asked about desired work percent the participants would prefer if the job was perfectly arranged for them, the intervention group responded with a mean of 72.7% (SD: 26.3), and the standard care group responded with a mean of 83.8% (SD: 22.6). No significant interaction effects were observed.

**Table 3:** Reporting of secondary feasibility metrics in terms of short-term effects.

Outcome measure	Group	Baseline	Week 6			Week 11			*p*-value for overall difference between groups^a^
		Mean (SD)	Mean (SD)	Adjusted mean difference between groups *β* (95% CI)^b^	*p*-value for adjusted mean d difference between groups	Mean (SD)	Adjusted mean difference between groups (95% CI)^c^	*p*-value for adjusted mean d difference between groups	
Primary outcome measures									
MS work difficulties questionare-23NV	CoreDIST	27.5 (11.0)	23.8 (13.7)	−3.1 (−9.2 to 3.0)	0.31	21.8 (13.2)	−3.9 (−8.8 to 1.1)	0.12	0.20
	Usual care	21.1 (10.9)	20.2 (13.4)			19.5 (12.0)			
6-min walk test (m)	CoreDIST	597.3 (72.6)	606.0 (81.7)	4.9 (−13.8 to 23.7)	0.59	611.9 (90.7)	17.9 (−9.4 to 45.2)	0.19	0.30
	Usual care	600.1 (74.4)	603.9 (69.8)			603.4 (60.1)			
Secondary outcome measures									
Mini-BESTest	CoreDIST	23.8 (1.9)	24.4 (1.2)	0.4 (−0.9 to 1.8)	0.50	25.3 (2.0)	0.4 (−0.7 to 1.6)	0.46	0.64
	Usual care	22.5 (2.5)	23.2 (2.7)			23.8 (2.7)			
Trunk Impairment Scale-modNV	CoreDIST	14.5 (1.5)	14.4 (1.5)	0.9 (−0.3 to 2.2)	0.14	14.5 (1.7)	1.0 (−0.4 to 2.4)	0.16	0.47
	Usual care	13.2 (1.9)	13.2 (3.1)			13.2 (2.4)			
AccuGait optimized for platform: RMS x-axis Eyes open (Anterior–Posterior, AP)	CoreDIST	1.0 (1.0)	0.8 (0.4)	−0.1 (−0.4 to 0.3)	0.74	1.0 (0.6)	0.03 (−0.4 to 0.5)	0.90	0.92
	Usual care	0.9 (0.5)	0.8 (0.5)			1.0 (0.8)			
AccuGait RMS x-axis Eyes closed AP	CoreDIST	1.0 (1.0)	0.8 (0.5)	0.1 (−0.3 to 0.4)	0.67	1.1 (0.7)	−0.1 (−0.5 to 0.4)	0.79	0.92
	Usual care	1.0 (0.7)	0.7 (0.5)			1.1 (1.0)			
AccuGait RMS y-axis Eyes open mediolateral (ML)	CoreDIST	3.3 (1.4)	3.3 (1.1)	0.04 (−0.8 to 0.9)	0.93	3.1 (1.2)	−0.3 (−1.1 to 0.6)	0.55	0.78
	Usual care	3.6 (1.3)	3.4 (1.2)			3.4 (0.9)			
AccuGait RMS y-axis Eyes closed ML	CoreDIST	3.5 (1.2)	3.0 (1.2)	−0.1 (−0.8 to 0.6)	0.74	2.9 (1.2)	−0.3 (−1.1 to 0.4)	0.37	0.47
	Usual care	3.4 (1.4)	3.1 (1.1)			3.2 (0.7)			
AccuGait RR- RMS x-axis (AP)	CoreDIST	1.2 (0.6)	1.0 (0.4)	0.1 (−0.2 to 0.3)	0.63	1.1 (0.6)	−0.1 (−0.5 to 0.3)	0.66	0.96
	Usual care	1.3 (1.0)	0.9 (0.4)			1.2 (0.6)			
AccuGait RR- RMS y-axis (ML)	CoreDIST	1.0 (0.2)	0.9 (0.2)	−0.1 (−0.2 to 0.1)	0.56	1.0 (0.3)	−0.02 (−0.2 to 0.2)	0.89	0.78
	Usual care	0.9 (0.2)	1.0 (0.3)			1.0 (0.3)			
ActiGraph number of steps per day	CoreDIST	9,269 (5,304)	9,524 (4,731)	978 (−724 to 2,680)	0.25	9,144 (4,262)	1,555 (161 to 2,950)	0.03	0.1
	Usual care	7,273 (3,148)	6,921 (3,357)			6,016 (2,445)			
ActiGraph activity level inactive (min)	CoreDIST	1,054 (76)	1,082 (80)	28.3 (−22.3 to 78.8)	0.26	1,089 (80)	−20.9 (−82.0 to 40.2)	0.49	0.79
	Usual care	1,084 (82)	1,085 (124)			1,136 (119)			
ActiGraph activity level Light (min)	CoreDIST	341.4 (49.5)	305.0 (63.8)	−36.8 (−80.2 to 6.7)	0.09	304.4 (78.8)	8.5 (−52.3 to 69.4)	0.77	0.49
	Usual care	323.0 (78.6)	321.8 (109.3)			279.1 (111.1)			
ActiGraph activity level Moderate (min)	CoreDIST	18.3 (14.3)	18.7 (15.5)	1.4 (−5.1 to 7.9)	0.66	17.3 (9.9)	5.0 (0.1–9.9)	0.05	0.24
	Usual care	15.9 (9.9)	15.3 (9.2)			10.9 (6.8)			
ActiGraph activity level Vigorous (min)	CoreDIST	27.2 (38.6)	35.8 (35.5)	9.2 (−2.6 to 20.9)	0.12	30.1 (32.6)	8.2 (−3.8 to 20.2)	0.17	0.12
	Usual care	18.8 (23.1)	20.2 (16.5)			15.7 (8.7)			
MS impact scale-29	CoreDIST	56.7 (16.5)	50.4 (17.5)	1.5 (−9.4 to 6.4)	0.71	52.0 (17.5)	−0.4 (−10.1 to 9.3)	0.94	0.94
	Usual care	51.8 (12.3)	47.8 (13.0)			47.8 (12.8)			
MS walking scale-12	CoreDIST	19.8 (9.9)	18.6 (8.7)	1.1 (−1.7 to 3.9)	0.42	20.6 (10.4)	4.2 (0.4–8.0)	0.03	0.06
	Usual care	20.8 (7.9)	18.2 (6.8)			17.0 (5.7)			
EQ-5D-3l	CoreDIST	0.7 (0.2)	0.7 (0.2)	−0.1 (−0.3 to 0.05)	0.16	0.8 (0.1)	−0.03 (−0.2 to 0.1)	0.62	0.28
	Usual care	0.7 (0.2)	0.8 (0.2)			0.8 (0.3)			

The results are demonstrated by means and standard deviation (SD) for the CoreDISTparticipation and usual care group, adjusted mean difference between the groups, 95% confidence intervals (CI), and *p*-values for the between-group differences at baseline and Weeks 6 and 11 measured by the primary and secondary outcomes. Linear mixed models were used to examine within-group differences over time and overall between-group differences at follow-up adjusted for baseline scores, where the term overall refers to the mean of the outcome values at Weeks 6 and 11.

^a^ Linear mixed model: Outcome (Weeks 6 + 11) = Outcome (Baseline) + Group.

^b^ Linear regression model: Outcome (Week 6) = Outcome (baseline) + Group.

^c^ Linear regression model: Outcome (Week 11) = Outcome (baseline) + Group.

At Follow-up one (6 weeks), all the remaining participants attended the assessments. One person only attended one (because of knee injury), and another attended no outdoor training sessions (because of illness). The same two individuals did not complete the 6MWT and the ActiGraph outcome measures at Week 11.

**Figure 2:**
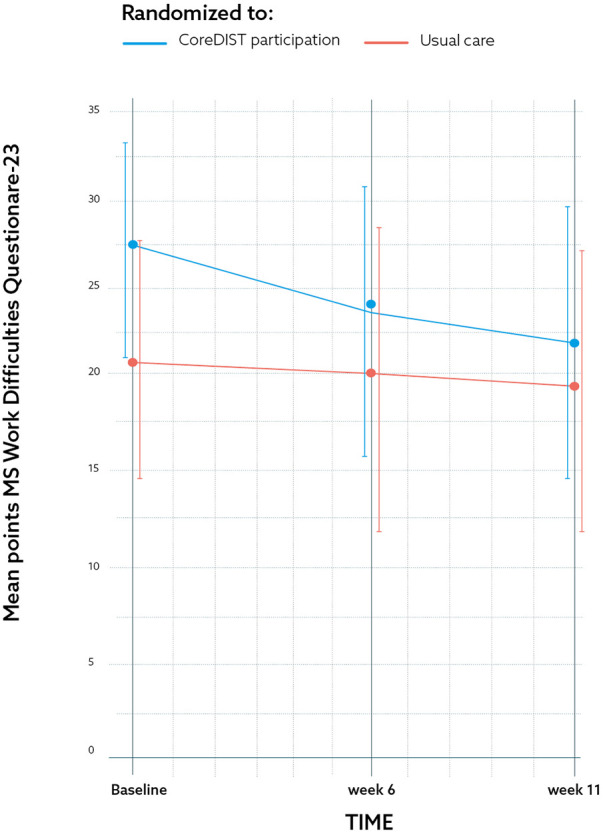
The results for one of the secondary feasibility metrics outcome measures; the Multiple Sclerosis Work Difficulties Questionnaire-23 Norwegian Version at baseline, Week 6, and Week 11 demonstrating CoreDISTparticipation (blue) and usual care groups (red) by mean and CI. The graph demonstrates a significant within-group 5.71-point improvement regarding barriers to work from baseline to Week 11 (*p* = 0.004) (CI: 2.17–9.25).

**Figure 3:**
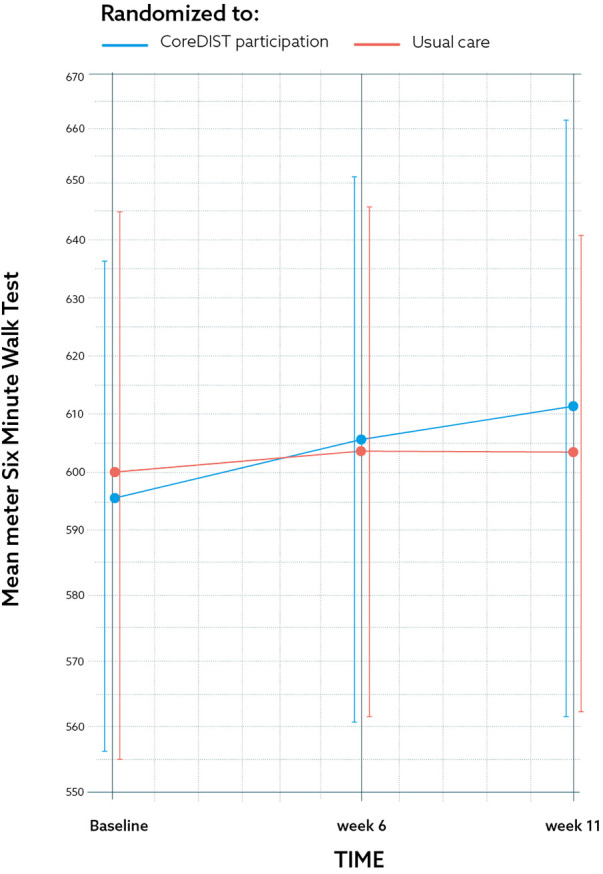
The results for one of the secondary feasibility metrics outcome measures; the 6-min walk test at baseline, 6, and 11 weeks for the CoreDISTparticipation group (blue) and usual care group (red) by mean and CI. The graph demonstrates a non-significant change of 14.6 m for the CoreDISTparticipation group from baseline to the 11-week retest.

The efficacy measures demonstrated no significant between-group differences between the CoreDISTparticipation and the control group. The results for the MSWDQ-23NV and 6MWT are presented in Figures 2, 3. The CoreDISTparticipation group demonstrated within-group changes in the MSWDQ-23NV [mean difference from baseline to Week 11 of 5.7 points, *P* = 0.004; confidence interval (CI): 2.2–9.3] and a non-significant tendency for change in the 6MWT from 597.3 m (SD: 72.6) at baseline to 611.9 m (SD: 90.7) at Week 11. Both the intervention (mean 14.5, SD: 1.5) and control group (mean 13.2; SD: 1.9) scored high at baseline on the TIS-modNV, which demonstrated a ceiling effect for this outcome (top score of 16). Mini-BESTest demonstrated within-group differences from baseline to Week 11 in both the CoreDISTparticipation (mean difference 1.5, *P* = 0.001, CI: −2.3 to −0.7) and the control group (mean difference 1.6, *P* = 0.003, CI: −2.5 to 0.7). The MSIS-29NV showed a within-group difference from baseline to Week 6 (mean difference 6.3 points, *P* = 0.006, CI: 2.1–10.5) in the CoreDISTparticipation group. Self-reported walking (MSWS-12), force platform, the EQ-5D-3l, and ActiGraphs demonstrated no changes. ActiGraphs revealed as much as 27.2 (38.6) min of vigorous intensity at baseline and a tendency for improvement, demonstrating 35.8 (35.5) min at Week 6.

Sample size calculation for a large-scale study is based on the mean score change from baseline to post-test at the MSWDQ-23NV (5.9 points for the intervention group and 2.3 for the control group). Assuming a common standard deviation of 6.0 for the score change, and calculating with a power of 0.8 and alpha = 0.05, the large-scale RCT will need to include 114 persons altogether (57 persons in each group) to detect a between-group difference in mean score change, assuming a dropout of 20%.

## Discussion

This study has identified primary feasibility metrics in relation to a new individualized, multidisciplinary intervention, termed *CoreDISTparticipation*. The study had a response rate to postal invitation of 34%, which is relatively low. There are many potential reasons why 66% of people invited did not respond, which could include: a lack of interest; self-assessment of not meeting the eligibility criteria; and a lack of sufficient time to potentially take part. In relation to employment, there is literature that indicates that 55%–70% of individuals living with MS are not employed (11–13), which may be a factor. The low response rate suggests that it would be potentially useful to consider alternative ways of recruiting potential candidates in future trials, for example, approaching charitable organizations who support people living with MS.

Despite the initial low response rate, after screening, the participant completion rate for the study was high: 100% (15/15) for the intervention group and 92.9% (13/14) for the usual care group. These high numbers demonstrated an interest in attending a comprehensive intervention. The attendance was high for the indoor GroupCoreDIST sessions (85%), which demonstrates feasibility and is in line with our previous CoreDIST studies (29, 33, 61). The moderate attendance in the outdoor sessions (57.3%) indicates needs for adjustments, even though most had good reasons for not attending (knee injury, illness). This was a period of the COVID-19 pandemic (although with no restrictions), and the low attendance is in line with pwMS reporting being less active during the pandemic (10, 30). The cold and rainy autumn weather may be another reason for not attending outdoor sessions. Another study from our research group however demonstrated that for those who attend outdoor training sessions, bad weather may provide a feeling of mastery (62). Further explorations of the outdoor environment may therefore be of interest. Three adverse reactions were reported during the high-intensity outdoor sessions. These are identified in Table 2 and were resolved by the municipal physiotherapist. The hamstring and ankle strains were treated acutely within the protection, rest, ice, compression, and elevation (PRICE) principle and advice. No further treatment was needed. The increased dizziness was assessed by the municipal physiotherapist and advice was provided. One person had a known heart condition and was worried about participating in the high-intensity training. This person was informed that high-intensity training is safe; however, chose to maintain a moderate intensity during the sessions**.** These are the relevant safety metrics for this trial. High-intensity interval training is reported to be safe and effective for pwMS with mild disability, but some studies do report some injuries (63). To increase safety during high-intensity exercise, aids, such as Nordic walking sticks, could be implemented in the intervention, and more alternatives for high-intensity exercise while standing could be offered. Exploring high-intensity activities in pwMS may be important for sustained function, as increased exercise capacity is indicated to influence neuroprotection and slow the rate of neuronal atrophy for pwMS (22, 24, 25). An intervention such as CoreDISTparticipation, which integrates high-intensity training with prerequisites for balance and walking, may therefore be an interesting contribution to the MS field.

The work-related follow-up was digital and had 100% attendance. Digital support opens new opportunities for the availability of competence and efficiency in communication between specialists and municipal healthcare. It is also a timesaving way of bringing employers into the loop. Good communication with the leader is one key for sustained employment (64). Such a multidisciplinary meeting may be a way of increasing communication, knowledge, and understanding (62). The meetings at the OP clinic with the MS nurse were however time-consuming, needed much logistics, and appear not to be feasible for a large-scale study. Instead, the welfare system will be linked to the intervention to address work in a more detailed manner within the existing system.

The necessary *resources* for this trial included the time needed for recruitment invitations to be posted, for clinicians to learn about the intervention, and assessors to learn about the selected outcome measures. The grant funding enabled adequate budgeting for the trial in terms of supporting the staff involved and access to the relevant equipment. All these metrics were adequate because of long-term and structured preparations and motivated clinicians and staff. For a potential larger multicentre study, it would be important to consider how to organize access to equipment for assessments at all site locations. With regard to overall trial *management*, it would be useful to consider increasing the size of the research group to speed up data management and review the equipment used for digital meetings that proved inadequate. Scientific aspects of the trial included evaluation of recordings, intervention content, dose, intensity, and the fidelity of the intervention and are further discussed.

Recordings demonstrated that the participants in both groups had mild disability (1.7 control/1.8 CoreDISTparticipation) and were young (mean 50.5 years in the usual care group/47.6 years in CoreDISTparticipation group), indicating the potential for a high work percentage. The rather low work percentage reported in both groups (65.4% usual care/46.3% CoreDISTparticipation) stands in contrast to their preferred work percentage if the job was perfectly adjusted to their needs, which was much higher (83.8% in usual care and 72.67% in CoreDISTparticipation). These results demonstrate a potential and desire to work more, which emphasizes the need to focus on employment in the follow-up of pwMS.

The results from the efficacy outcome measures demonstrated no between-group differences, as expected in a pilot study. However, there were within-group differences for the primary outcome MSWDQ-23NV, with a mean improvement of 5.9 points reduction of barriers for work at Week 11 in the CoreDISTparticipation group. This indicates that integrating work, physical activity, and sensorimotor function may have a potential impact on barriers to work in pwMS and demonstrates the feasibility of the MSWDQ-23NV as a primary outcome in a future study. The significant within-group change in HRQoL (MSIS-29NV) may support the decreased barriers for work, as some of the items in MSIS-29NV are related to items in the MSWDQ-23NV. However, the changes of over 6 points in the MSIS-29NV did not meet the 8 points needed for a clinically meaningful difference (56). The MSIS-29NV seems suitable for detecting changes in a future large-scale study. Fatigue was only measured through the elements in the mentioned outcomes and should be included in a larger study, as fatigue is a common reason for unemployment and because exercise may reduce fatigue in pwMS (21, 65).

The 6MWT and the physical activity monitors (ActiGraphs) demonstrated no significant change in the distance walked over 6 min, number of steps, or physical activity levels. Interestingly, the participants in the CoreDISTparticipation group walked a high mean number of steps already at baseline [mean 9,269 (SD: 5,304)] and first retest [9,524 (SD: 4,731)]. They also recorded more minutes in vigorous physical activity than expected, with a mean of 27.2 min (SD: 38.6) at baseline and 35.8 min (SD: 35.5) at the 6-week retest. Current physical activity recommendations (23) were, in contrast to most other studies (10, 29, 66, 67), fulfilled in our sample. The high levels of physical activity may indicate a biased sample of very active pwMS, or at least that a few persons who were particularly active biased the results in this small sample. No significant improvements in physical activity are in line with other studies, and it is well documented that changing physical activity habits is a challenge (68). Some studies emphasize the success criteria for sustainability, self-mastery, and long-term behavioural change of physical activity include a long-term follow-up, behavioural change techniques, and activity choices (68–70), and these elements may be considered integrated in a future study. Quite few RCT studies measure physical activity objectively, and studies that measure physical activity are warranted (69).

The within-group changes demonstrated at the Mini-BESTest may be due to the detailed sensorimotor and balance exercises undertaken during the in- and outdoor sessions and the walking/running in various terrains. The indoor sessions address prerequisites for postural control and balance, such as somatosensory function, muscle length, trunk control, larger muscle groups, and selective movements, and the outdoor sessions address the activity itself. Both are of importance for optimizing anticipatory postural adjustments: the minor adjustments in the trunk, hips, and ankle/legs that prepare for predictable perturbations of the COP before and during any movement (71), and compensatory postural adjustments that are used to regain balance after unpredictable perturbations of the COP (72). In an outdoor setting, dual/multiple tasks are additionally needed constantly, which may also be captured through the Mini-BESTest. The TIS-modNV demonstrated a ceiling effect and was considered removed from a large-scale study. However, in a different sample, trunk control may be more prominent, as trunk control may also be affected in those with mild to moderate disability (73). Trunk control is a central element in the CoreDIST intervention and should be assessed in a future study. Furthermore, objective measures of balance through the force platform should be further explored, as standing on two legs with eyes open and closed did not reveal any change. More challenging postural control tasks, such as one-leg standing, may be relevant to add to this mildly disabled population.

### Limitations

A significant limitation to this study is the lack of identification of what usual care was. This means that there can be no clear conclusion or comparisons drawn between the two groups because there is no documented information on what the usual care standard was. In addition, the sample size of the study was small, though adequate in terms of feasibility. For this reason, caution is recommended regarding the interpretation of secondary effects. A further limitation is the higher percentage of women than men participating, though this does correspond to the gender distribution in the MS population (1). To improve the understanding and accuracy of potential future intervention implementation, it would be beneficial for participants to record and document exercise choice and number of repetitions performed within sessions, as well as individualization. The intensity was only partly measured by pulse belts and watches, and these recordings should be conducted in detail in a future study. An 11-week follow-up is relatively short, and a longer-term follow-up would be beneficial.

## Conclusion

This study demonstrated that it is feasible and safe to use the CoreDISTparticipation intervention to support pwMS who live in the community. While there were no statistically significant differences in the clinical outcome measurements taken between the two groups at the end of the trial, some within-group effects for the CoreDISTparticipation group regarding barriers for work, HRQoL, and balance were found. It could be interesting to consider a larger future trial with a detailed recording of the usual care, feasibility metrics, and implementation of overall trial reflections and learning.

## Data Availability

The datasets presented in this article are not readily available because the dataset is at the moment not available as there is no current permission from the ethical committee to share data. Requests to access the datasets should be directed to ellen.c.arntzen@nord.no.

## Appendix. Description of the CoreDISTparticipation intervention

**Table:**

Period	CoreDISTparticipation intervention
(1) A: 40 min and B: 60 min	(A) A digital meeting with the MS nurse: a structured conversation addressing work-related issues and possible needs for adaptations by going through the MSWDQ-23NV. Goals regarding potential elements at work that need to be improved were set.
	(B) A clinical assessment by the physiotherapist aiming to explore underlying sensorimotor prerequisites causing balance and/or walking challenges, and possibilities for change in these. Information regarding the findings was sent to the municipal physiotherapist.
(2) *Municipality Part 1*: (Weeks 2–5) 60 min two times per week for 4 weeks Home training was encouraged two times per week for 30 min.	(A) A physiotherapist continued to explore the participants’ possibilities for improvements in sensorimotor function and balance, explore CoreDIST exercises, and identify goals for physical activity for the following 8 weeks.
	(B) Four weeks with indoor training two times per week for 60 min using GroupCoreDIST exercises (31) in groups of 3–5 persons, focusing on trunk control and other prerequisites for balance and postural control such as somatosensory function, muscle length, trunk control, larger muscle groups, and dual task as well as encouragement for increased physical activity.
	(C) To promote participation in employment, the standardized questionnaire regarding barriers for work MSWDQ-23NV and a self-created screening form regarding barriers and facilitators for physical activity were sent by email to the participants and their leaders at work, encouraging them to have a conversation regarding facilitators and barriers at work and physical activity as well as setting goals for the 8-week period.
	(D) To promote both participation in employment and physical activity, a structured digital meeting including the pwMS, their employer, the MS nurse, and the municipal physiotherapist was conducted.
	(E) The participants had access to seven different 10–15-min-long home training videos (free and available at www.nord/CoreDIST), which they were encouraged to follow at home or at work, minimum two times per week.
(3) *Municipality Part 2*: (Weeks 7–10) 60 min two times per week 4 weeks Home training was encouraged two times per week for 30 min.	(A) The following four weeks consisted of outdoor CoreDIST balance and high-intensity training two times per week for 60 min in larger groups (up to 10 persons). This part of the intervention called CoreDIST-stamina and strength combined CoreDIST exercises with running or walking. The session was structured with 20 min warm up with exercises followed by 3–4 intervals of 4 min with high-intensity and CoreDIST exercises. The running/walking was organized in a self-developed system named “the star”. The participants picked up pea-bags in the exercise area and ran towards cones placed at various distances (10–100 metre) and in various terrains (asphalt, grass, gravel, flat, uphill, and downhill). After completing an interval, the participants counted their pea-bags and were encouraged to repeat the same number of pea-bags for the next round. In some rounds, dual tasks were added as the participants were encouraged to add up the numbers printed on their pea-bags (printed numbers from 1 to 100). The benefits of physical activity were addressed in the cool-down after the intervals. Some participants wore pulse belts and watches during the outdoor training to record the intensity of the training.
	(B) The seven home training videos were sent one at a time after each outdoor session to the participants’ smartphones, encouraging them to follow a particular program before the next session.
	At the end of the period, the MSWDQ-23NV and a standardized physical activity form were sent out to the participants encouraging them to talk to their leaders regarding goal achievement.
